# Maintaining Gait Performance by Cortical Activation during Dual-Task Interference: A Functional Near-Infrared Spectroscopy Study

**DOI:** 10.1371/journal.pone.0129390

**Published:** 2015-06-16

**Authors:** Chia-Feng Lu, Yan-Ci Liu, Yea-Ru Yang, Yu-Te Wu, Ray-Yau Wang

**Affiliations:** 1 Translational Imaging Research Center, College of Medicine, Taipei Medical University, Taipei, Taiwan, ROC; 2 Department of Radiology, School of Medicine, Taipei Medical University, Taipei, Taiwan, ROC; 3 Department of Physical Therapy and Assistive Technology, National Yang-Ming University, Taipei, Taiwan, ROC; 4 Taipei City Hospital, Taipei, Taiwan, ROC; 5 Institute of Biophotonics, National Yang-Ming University, Taipei, Taiwan, ROC; 6 Department of Biomedical Imaging and Radiological Sciences, National Yang-Ming University, Taipei, Taiwan, ROC; 7 Brain Research Center, National Yang-Ming University, Taipei, Taiwan, ROC; Duke University, UNITED STATES

## Abstract

In daily life, mobility requires walking while performing a cognitive or upper-extremity motor task. Although previous studies have evaluated the effects of dual tasks on gait performance, few studies have evaluated cortical activation and its association with gait disturbance during dual tasks. In this study, we simultaneously assessed gait performance and cerebral oxygenation in the bilateral prefrontal cortices (PFC), premotor cortices (PMC), and supplemental motor areas (SMA), using functional near-infrared spectroscopy, in 17 young adults performing dual tasks. Each participant was evaluated while performing normal-pace walking (NW), walking while performing a cognitive task (WCT), and walking while performing a motor task (WMT). Our results indicated that the left PFC exhibited the strongest and most sustained activation during WCT, and that NW and WMT were associated with minor increases in oxygenation levels during their initial phases. We observed increased activation in channels in the SMA and PMC during WCT and WMT. Gait data indicated that WCT and WMT both caused reductions in walking speed, but these reductions resulted from differing alterations in gait properties. WCT was associated with significant changes in cadence, stride time, and stride length, whereas WMT was associated with reductions in stride length only. During dual-task activities, increased activation of the PMC and SMA correlated with declines in gait performance, indicating a control mechanism for maintaining gait performance during dual tasks. Thus, the regulatory effects of cortical activation on gait behavior enable a second task to be performed while walking.

## Introduction

In daily life, mobility requires walking while performing a second task. The ability to perform 2 tasks concurrently (dual task) enables people to communicate with others, use mobile phones, transport objects, and respond to environmental stimuli when walking. Gait is considered an automated motor task and involves higher-level cognitive functions, such as attention [1, 2] and executive function [3, 4]. When a person performs a second cognitive or motor task when walking, increasing demands in cognitive coordination or posture adjustment result in effects on gait performance and brain activities.

Previous studies have investigated the effects of dual tasks on gait performances in healthy adults, observing reduced gait speed and stride length, and increased cadence [1, 5–8]. Although these studies elucidated some effects of dual tasks on gait performance, brain activities when performing dual tasks and their association with gait disturbance are not well described. Recently, several studies reported prefrontal activations during gait dual-tasking for healthy adults. Atsumori et al (2010) described increased activation of the prefrontal cortex (PFC, predominantly right side) when walking and balancing a ping pong ball on a small card in healthy young adults [9]. The aging effect on reducing the bilateral PFC activations was observed during walking while talking [4], walking with a complex visual task [10], and walking while performing serial 7 subtractions [11]. Mirelman et al (2014) further proposed that the rostral PFC activation during walking while carrying out serial 7 subtraction is not solely a reflection of cognitive demands in mental calculation for young adults [12]. These studies (excluding Atsumori (2010)) also measured the gait performance, such as gait speed, stride time, stride length, and gait variability. Simultaneous assessments of gait and brain activities can be helpful to determine the mechanisms involved in brain manipulation of locomotive behaviors when walking and performing a second task.

The effect of the concurrent task type on locomotion has also been established in previous studies. Different demands of the second task lead to different levels of dual-task interference ranging from 0.99 to 26.0% declines in gait for young adults [13–15]. The theory of multiple resource models suggests that the dual-task interference is minimal if 2 tasks use differing functional resources, [16, 17]. In contrast, performing 2 tasks with similar cognitive or motor demands can cause retardations in both tasks or delays in the secondary task based on the capacity-sharing theory and bottleneck theory [18, 19]. Another factor of dual-task interference is the complexity or the difficulty of the concurrent task [20, 21]. A more complex cognitive task requiring higher attention and processing resources over walking, and therefore interferes with gait to a greater extent. In this study, we conducted a cognitive- and a motor-oriented dual task to observe the dual-task interference in gait and the induced brain activations from these 2 different dual tasks.

Previous studies on brain activation during gait were limited by the majority of available brain imaging tools being unsuitable for analyzing locomotion. For example, functional magnetic resonance imaging requires participants to be scanned in a supine posture with a fixed head position. Alternatively, electroencephalography allows participants to perform locomotive tasks, but the physiological and mechanical noise (such as from blinking, facial-muscle movements, electrode movements, and high-frequency signals from the treadmill) need to be eliminated by using post-processing techniques to avoid the interference with signal recordings of neural activity [22, 23]. Another recently developed functional near-infrared spectroscopy (fNIRS) technique can also be used for observing cortical activation during locomotion [24–28]. Similarly, relevant motion correction techniques for fNIRS signals are demanded to effectively remove the three categories of fNIRS motion artifacts, namely, spikes, baseline shifts and low-frequency variations, during motion tasks [29, 30].

Although previous dual-task studies predominantly focused on activation in the PFC, the premotor cortex (PMC) and supplemental motor areas (SMA) are involved in adapting walking speed and posture [24, 27, 31]. We anticipated that walking while performing a second task alters gait patterns, such as by reducing speed and increasing cadence. Therefore, in this study, we measured cortical activity in the PMC, SMA, and PFC to investigate movement planning and postural control of locomotion during dual-task walking. We simultaneously evaluated cerebral activation (by using fNIRS) and gait performance when participants walked and performed a cognitive or motor task to determine whether changes in brain activity correlate with gait alterations during dual tasks.

The specific aims of this study were to evaluate (1) the declines in gait performance caused by differing dual-task interference; (2) the alterations in cortical activation in the PFC, PMC, and SMA when walking and performing a second cognitive or motor task compared with walking at a normal pace; and (3) the association between cortical activation and gait performance during dual tasks. We hypothesized that the cortical activation in the PFC, PMC, and SMA may increase due to the additionally cognitive or motor demand when walking and performing a second task. Furthermore, the association between cortical activation and gait performance during dual tasks can be statistically identified and used to determine the control mechanisms for bipedal movement with dual-task interference.

## Methods

### 2.1 Participants

Seventeen healthy participants (9 men and 8 women) with an average age of 23.1 ± 1.5 years were recruited. This study received prior approval from the Institutional Review Board of Taipei Veterans General Hospital and each participant provided written informed consent. All participants were right-handed with no history of neurological or physical disorder, alcohol or drug dependence, or ataxia.

### 2.2 Walking paradigm

The walking paradigm included conditions of walking at a normal pace and walking and performing a second cognitive or motor task. Brain hemodynamics and gait data were recorded simultaneously during tasks.

- Normal-pace walking (NW): Participants were asked to walk at their normal pace on a walkway (approximately 5.50 m long and 0.90 m wide) from one end to another in a quiet room, and to turn at the end of walkway and continue walking (for 60 s) until instructed to rest.
- Walking while performing a cognitive task (WCT): Participants were asked to walk on the same walkway while serially subtracting 7 from an initial 3-digit number and speaking out each calculated number as quickly as possible. Participants were asked to focus on 7 serial subtractions. The task period was approximately 60 seconds and ended with a resting instruction.
- Walking while performing a motor task (WMT): Participants were asked to walk on the same walkway while carrying a 600-mL bottle of water on a tray. Participants were asked to avoid dropping the bottle while walking. The task period was approximately 60 seconds and ended with a resting instruction.

A block design with a random order of the 3 walking conditions was applied. Each walking condition was repeated 3 times to yield a total of 9 walking blocks (block length 60 s). The experiment began with a fixed standing condition (for 60 s), followed by one of the 3 walking conditions and then a resting condition (for 60 s). During the resting condition, participants were asked to sit comfortably on a chair and then to stand stationary at the end of the walkway for 15 seconds before the subsequent walking condition. Before each walking task, audio instruction was provided to inform participants of the type of walking task to be performed.

### 2.3 Gait performance

Quantitative gait analyses were conducted using an electronic walkway system (GAITRite, CIR system, Inc, Havertown, PA, USA, 4.75 × 0.89 m) with sensor pads connected to a laptop computer [32, 33]. When a participant walked along the walkway, the contact time and location of each footfall were recorded and analyzed using the application software. The participants were asked to turn outside the area of the electronic walkway system; therefore, gait data during turning were not recorded. To increase measurement reliability, gait data were recorded for all blocks of each walking condition and averaged separately for each walking condition [33]. Five gait properties, including speed, cadence, stride time, stride length, and gait variability were analyzed. Gait speed is defined as the walking distance in a second (in cm/s); cadence is defined as the number of steps within a minute walk (in steps/min) [34]; stride time and stride length indicate the time interval (in second) and the distance (in cm) from initial contact of one foot to subsequent contact of same foot, respectively [35]; and gait variability is calculated as the coefficient of variation (= standard deviation/mean x 100%) of the stride time [36, 37].

### 2.4 Monitoring cerebral activity using functional near-infrared spectroscopy

**Fig 1:**
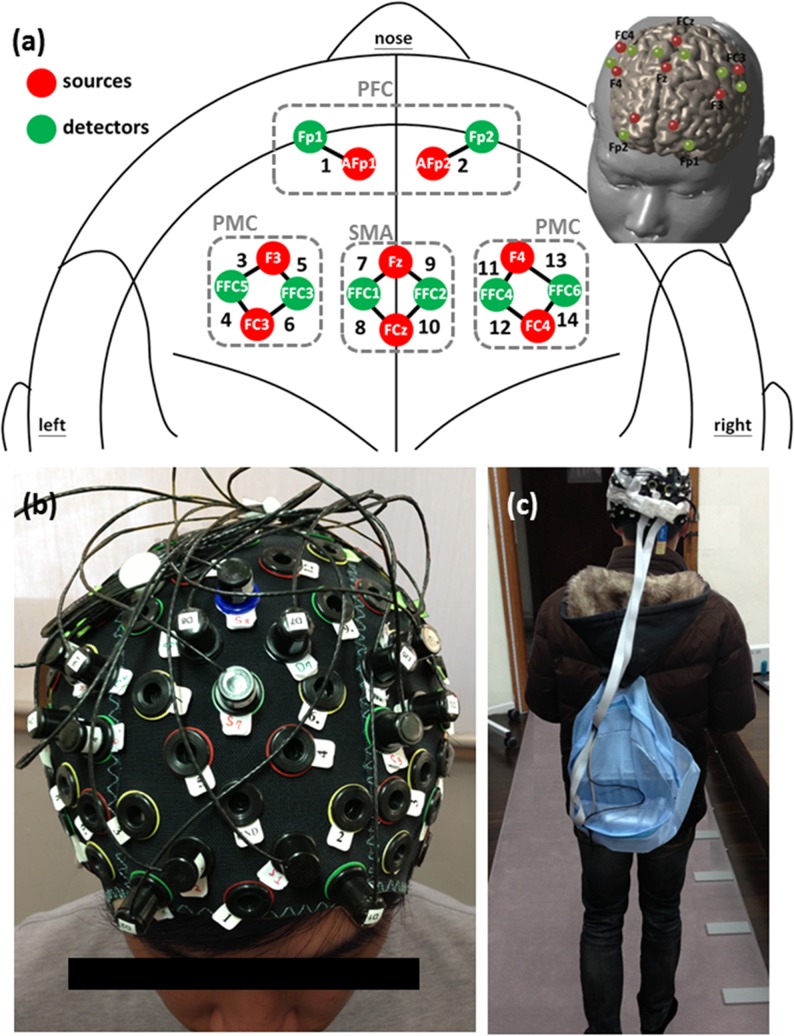
The locations of fNIRS channels and the experimental environment. (a) The arrangement of source-detector locations based on the international 10–5 system. Red circles stand for the source locations and green circles for the detector locations. Each pair of source and detector with distance of 3.0 cm can form an effective channel (presented by the black bold line between optodes and numbered by black digits), resulting in totally 14 channels distributed over bilateral PFC (Channels 1 and 2), left PMC (Channels 3 to 6), bilateral SMA (Channels 7 to 10), and right PMC (Channels 11 to 14); (b) The setup of fNIRS optodes (8 sources and 8 detectors) on a participant’s head; (c) A demonstration of the experimental environment. The participant wears a backpack with the fNIRS control box and a connected laptop for data acquisition inside (approximately 1 kg) and walks on a sensor-embedded walkway.

A multichannel wearable fNIRS imaging system (NIRSport, NIRx Medical Technologies LLC, Glen Head, NY, USA) was used to simultaneously acquire dual-wavelength (760 and 850 nm) signals. The fNIRS optodes, including 8 LED light sources and 8 detectors, were attached to participants’ heads to monitor the hemodynamics of the bilateral PFC, PMC, and SMA (Fig 1A). The fNIRS head cap was designed to be compatible with the international 10–5 system, which defines standard surface positions for a human head with approximately 3.0 cm between any 2 adjacent positions (Fig 1B) [38]. In the source and detector arrangement, 14 source-detector channels were used for monitoring local blood oxygenation (Fig 1A), with a sampling rate of 7.81 Hz. The fNIRS control box and a connected laptop computer for data acquisition were placed in a backpack worn by participants (Fig 1C). The overall fNIRS system weighs approximately 1 kg, which exerts minimal influence on gait performance. During the signal acquisition, we also recorded the time points for the turning phases (walking outside the electronic walkway system) and excluded the signals within the turning phases from the subsequent statistics and correlation analyses.

To validate the locations of the fNIRS channels, a procedure of coregistration between the source and detector locations and the structural T1-weighted magnetic resonance (MR) image, using anatomical landmarks, was conducted for each participant [4]. Specifically, the anatomical landmarks (including nasion and bilateral pre-auricles) and surface locations of the fNIRS optodes were recorded using a 3-dimensional (3D) digitizer with ultrasound transmitters and sensors (CMS20 measuring system, Zebris Medical GmbH, Germany). T1-weighted images were acquired using a 3D fast-spoiled gradient echo pulse sequence on a 3T MR scanner (MAGNETOM Trio, A Tim System 3T, Siemens, Germany). The imaging parameters were as follows: repetition time = 2530 milliseconds, echo time = 3.03 milliseconds, flip angle = 7°, field of view = 224 × 256 × 192 mm^3^, and voxel size = 1 × 1 × 1 mm^3^. The surface locations of fNIRS optodes can be transferred to the same coordinates, with structural MR images based on the corresponding anatomical landmarks, using the iterative closest point algorithm [39]. S1 Fig of the supplemental material shows the coregistration procedure.

### 2.5 Brain hemodynamic signals

To estimate the signal-to-noise quality of a data channel, the relative coefficient of variation (CV, in %) was calculated for the raw signals at 760 and 850 nm, which is a routine procedure for fNIRS measurement [40, 41]. Data rejection based on 2 types of CV, CV_chan_ and CV_trial_, was used to reduce physical artifacts such as motion-induced instabilities and blood pressure-induced hemodynamics [42].

$$CV=\frac{\sigma }{\mu }\times 100\%$$

where μ is the mean value and σ is the standard deviation of the signal. CV_chan_ was calculated over the entire duration of the experiment (approximate 19 min) for each channel, and measurement channels with CV_chan_ > 15% were rejected. The CV_trial_ was then obtained for 60-second intervals of the individual trial block, and only trials for each remaining channel (CV_chan_ < 15%) with CV_trial_ < 10% in both wavelengths were retained for subsequent analyses. The average rejection rate for channels per participant was 2.1% with a standard deviation (SD) of 4.9%, and the average rejection rate for trials per channel was 0.5% with an SD of 3.4%.

The remaining fNIRS signals were bandpass-filtered (low-cutoff frequency 0.01 Hz and high-cutoff frequency 0.2 Hz) to eliminate the effects of heartbeat, respiration, and low-frequency signal drifts for each wavelength [42–44]. The principal component analysis (PCA) [45] and spike rejection [30, 46] were subsequently used to correct for the motion artifacts. An adaptive PCA were applied to the filtered fNIRS signals based on the CV_trial_ of signals. Specifically, a removal of 80% signal variance was employed for the signal periods with CV_trial_ smaller than 5% and a removal of 97% variance for the periods with CV_trial_ between 5% and 10% to effectively correct the larger signal disturbance caused by motion artifacts and preserve the possible evoked hemodynamic response [30, 45, 47, 48]. Signal spikes or dramatic changes in signal amplitude are usually produced by a sudden small cap shifts and are difficult to be corrected by the present methods. These spike artifacts can be identified and eliminated by using the *hmrMotionArtifact* algorithm in the HOMER2 fNIRS processing package [46]. The preprocessed signals were converted to concentration changes in oxygenated hemoglobin (HbO) and deoxygenated hemoglobin (HbR) using the modified Beer-Lambert law for each source-detector channel [49–51]. The relative changes in HbO and HbR concentrations for each walking condition were obtained using a 5-second baseline (approximate 40 frames) collected before each task block. Finally, the HbO and HbR changes were averaged over 3 repetitions for each walking condition to improve signal-to-noise ratio [42, 51]. The fNIRS signal preprocessing, including the motion artifact correction, bandpass filtering, and conversion of HbO and HbR were processed using the HOMER2 package [46]. The calculation of signal CVs and correlation analyses were performed using home-made scripts developed on MATLAB (Mathworks, Natick, MA, USA).

### 2.6 Brain activation during early and late phases when performing tasks

Neuronal activation typically induces a rapid increase in HbO and a lower-amplitude reduction in HbR based on neurovascular coupling, to result in increased local oxygenation [31, 52–56]. An index of hemoglobin differential (Hbdiff = HbO–HbR) was used to evaluate brain activation [4, 57–59]. Changes in brain activation were analyzed statistically using a one-sided *t* test against zero to determine whether oxygenation increased significantly during task blocks.

**Fig 2:**
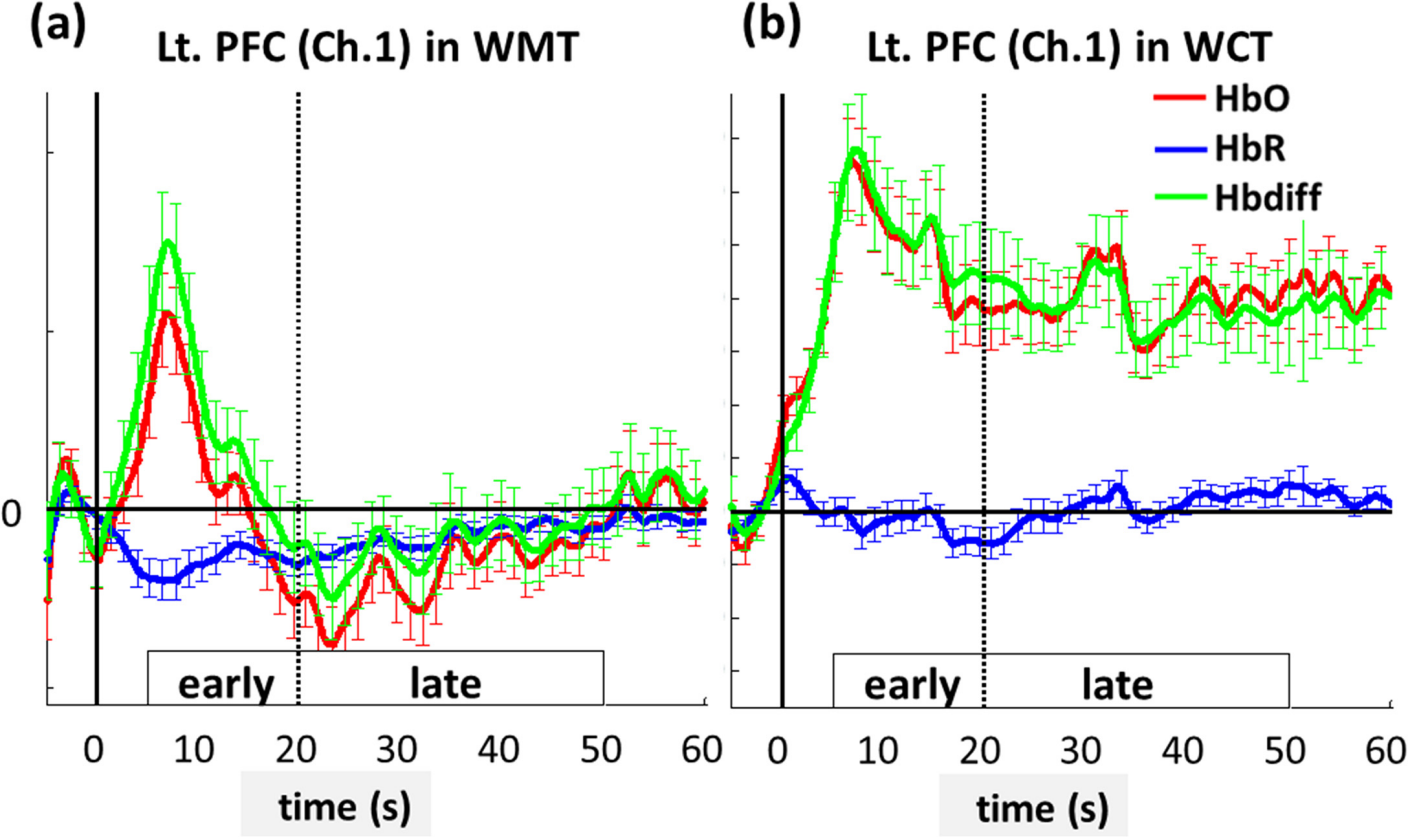
The averaged dynamics of HbO (red curves), HbR concentrations (blue curves), and Hbdiff level (green curves) at the left PFC (Channel 1) during (a) WMT and (b) WCT. The horizontal solid lines depict the concentration level of zero, and the vertical solid lines label the time of zero for the task onset. The early and late phases are defined as the periods of 5 to 20 s and 21 to 50 s after the task onset, respectively. The error bars represent the corresponding standard errors of mean.

A block design with a 20–30-second task period is commonly used in fNIRS studies [9, 42, 56, 60]. In this study, a longer task period (60 s) was used to investigate brain activation in the first 20 seconds after task onset as well as in the subsequent 30 seconds. The period between 5 and 20 seconds after task onset was defined as the early phase to reflect the immediate hemodynamic response when performing tasks (Fig 2). The period between 21 and 50 seconds after task onset was defined as the late phase to assess continuous brain activation (Fig 2). The 3 possible patterns of activation were no activation in early and late phases, activation in the early phase but not the late phase (Fig 2A), and activation in early and late phases (Fig 2B). The first 5 seconds and final 10 seconds of each task period were excluded to eliminate the transient periods of hemodynamic responses [25].

### 2.7 Statistical and correlation analysis

Gait data, including walking speed, cadence, stride time, and stride length, were evaluated separately using a repeated one-way ANOVA with the null hypothesis that gait measurements in 3 tasks are collected from populations with the same mean values. When a difference between 3 tasks was detected (*P* < .05), posthoc testing of 3 pairwise comparisons (WCT vs NW, WMT vs NW, and WCT vs WMT) was conducted using paired *t* tests with Bonferroni correction for multiple testing (*P* < .05/3 = .016).

Brain activation during each walking condition and phase was identified as a significant increase in Hbdiff by performing a one-sided *t* test against zero (*P* < .025) with false discovery rate (FDR) correction of multiple comparisons for 14 channels [61]. A repeated one-way ANOVA was then used to test the null hypothesis that the Hbdiff in the 3 tasks are collected from populations with the same mean values for each channel. Posthoc testing of 3 pairwise comparisons of cortical Hbdiff, WCT vs NW, WMT vs NW, and WCT vs WMT, was then conducted using paired *t* tests (*P* < .05) with the FDR correction of multiple comparisons for 14 channels and 3 conditions.

The relationships between cortical Hbdiff and gait data were examined for each channel in different phases by calculating partial correlation coefficients and controlling for age and sex as the confounding variables. Each observation of the correlation analysis was the measurement from a single task block, resulting in approximate 51 observations (17 subjects*3 *blocks*–*bad trials*) for each walking condition. Significant correlations were defined as *P* < .05 with FDR correction for the multiple correlations.

## Results

### 3.1 Alterations in gait performance during dual tasks

Table 1 lists our results on gait performance in the various walking conditions. Participants walked significantly slower during WCT than during NW (10.03 cm/s slower). During WCT, reduced walking speed was caused by reductions in cadence (of 4.38 steps/min) and stride length (by 3.30 cm). Stride time was an average of 0.03 seconds longer during WCT than during NW.

**Table 1:** Statistics of gait performance between 3 walking conditions.

Gait data	NW	WCT	WMT	Statistical results	*p*-value*
**Speed**	112.69±11.90	102.66±11.23	103.98±12.35	NW > WCT	< 0.0001
(cm/second)				NW > WMT	0.0033
				WCT = WMT	0.5415
**Cadence**	114.56±6.22	110.19±6.51	115.70±6.25	NW > WCT	< 0.0001
(steps/min)				NW = WMT	0.3855
				WCT < WMT	< 0.0001
**Stride time**	1.06±0.06	1.09±0.06	1.04±0.05	NW < WCT	0.0040
(second)				NW = WMT	0.2153
				WCT > WMT	< 0.0001
**Stride length**	118.58±10.72	112.28±10.49	108.45±11.10	NW > WCT	< 0.0001
(cm)				NW > WMT	< 0.0001
				WCT = WMT	0.0427
**Gait variability**	2.69±0.85	2.38±1.01	2.94±1.02	NW = WCT	0.3196
(%)				NW = WMT	0.3525
				WCT = WMT	0.1172

*The significance was defined as *p*<0.016 (Bonferroni correction for multiple testing).

Our results indicated that the reductions in walking speed during WMT were similar to those during WCT; however, the gait properties (cadence and stride time) differed significantly between the 2 dual-task types. Participants walked a greater number of steps (higher cadence) with a shorter stride time during WMT than during WCT (Table 1). Therefore, only the walking speed and stride length decreased significantly during WMT compared with during NW (Table 1). No significant difference in gait variability was found between 3 walking conditions (Table 1).

### 3.2 Patterns in brain activation during dual tasks

**Fig 3:**
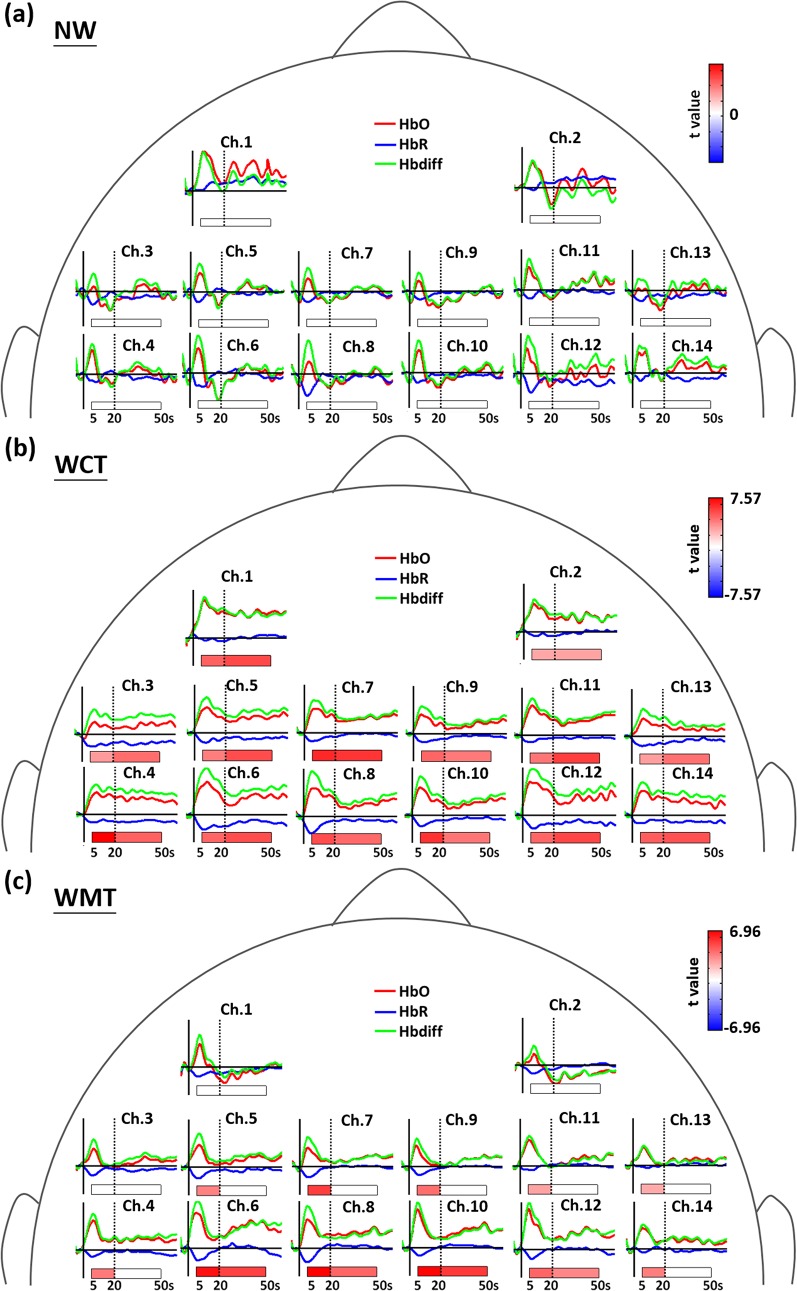
The statistical results in brain activation during (a) NW, (b) WCT, and (c) WMT, respectively. The *t* values of significant activations with FDR correction for the multiple comparison in early or late phase are color-coded under the axis for each channel. The averaged dynamics of HbO (red curves), HbR concentrations (blue curves), and Hbdiff level (green curves) are also displayed at each channel. The horizontal solid lines depict the concentration level of zero, and the vertical solid lines label the time of zero for the task onset.

As shown in Fig 3A, we observed nonsignificant brain activation in the early and late phases of NW. Although Hbdiff tended to increase in early-phase NW in the majority of channels, the changes were nonsignificant after FDR correction for multiple comparisons. By contrast, we observed significant brain activation during both types of dual task. During WCT, all 14 channels in the PFC, PMC, and SMA were associated with sustained increments in Hbdiff during the entire task period (Fig 3B). We observed strongest activation (largest *t* values) in the left PFC (Channel 1) in early and late WCT phases. During WMT, 10 of 14 channels, excluding the bilateral PFC (Channels 1 and 2) and parts of the bilateral PMC (Channels 3 and 13), were associated with significant increases in Hbdiff in the early phase (Fig 3C). Several channels in the bilateral SMA (Channels 8 and 10) and PMC (Channels 4, 5, 6, and 12) exhibited continuous activation in late-phase WMT. We observed strongest activation (largest *t* values) in the right SMA (Channel 10) in both WMT phases.

### 3.3 Patterns of brain activation in different walking conditions

**Fig 4:**
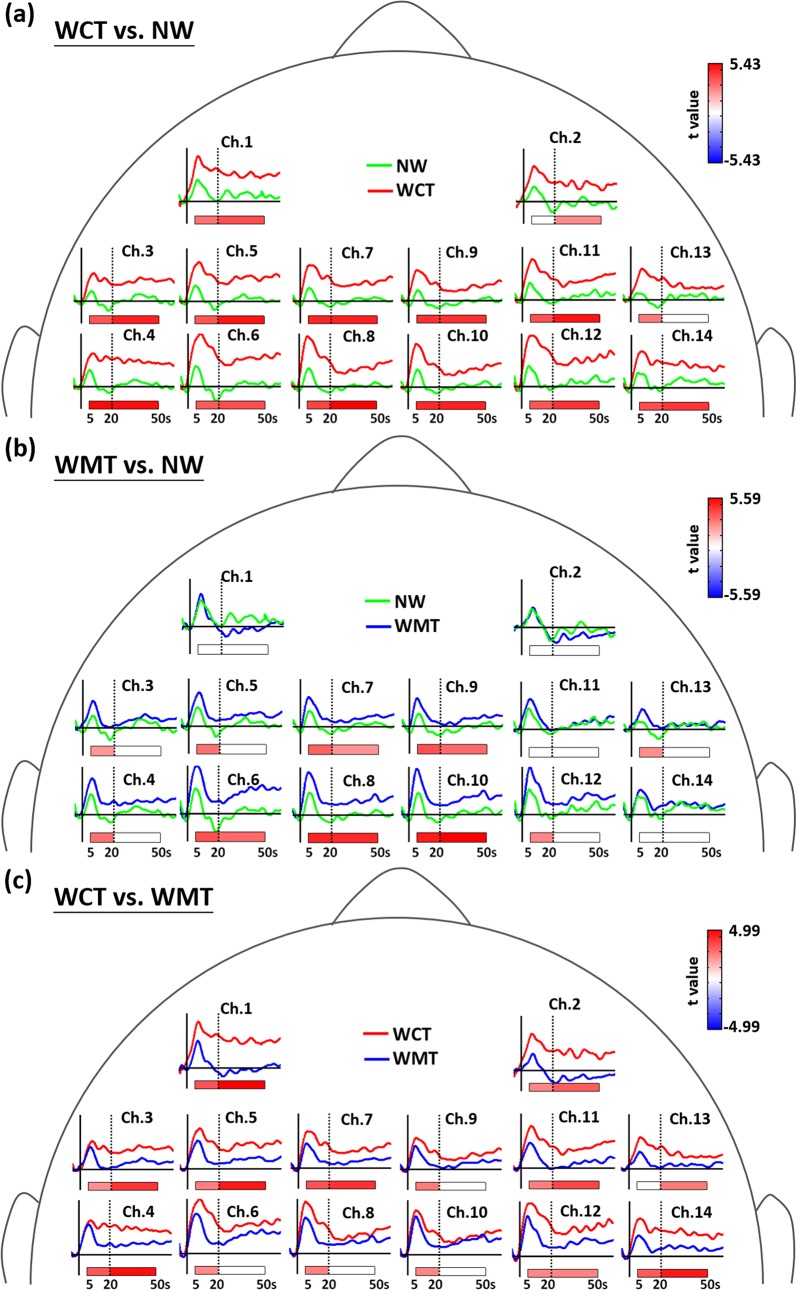
The statistical results in difference of brain activation for the three comparisons of (a) WCT vs. NW, (b) WMT vs. NW, and (c) WCT vs. WMT. The *t* values of significant differences with FDR correction for the multiple comparison in early or late phase are color-coded under the axis for each channel. The averaged dynamics of Hbdiff level for different tasks (green for NW, red for WCT, and blue for WMT) are also displayed at each channel. The horizontal solid lines depict the concentration level of zero, and the vertical solid lines label the time of zero for the task onset.

Our pairwise comparison statistical results revealed differing patterns of brain activation among the 3 walking conditions. The majority of channels presented with higher Hbdiff during early and late phases of WCT than they did during NW (Fig 4A). Channels in the SMA (Channels 7–10) and left PMC (Channels 5 and 6) also exhibited stronger activation during early and late phases of WMT than they did during NW (Fig 4B). When we compared the 2 dual-task types, we observed that only the channel in the right SMA (Channel 10) exhibited nonsignificant differences in Hbdiff in both early and late phases (Fig 4C). Thirteen channels in the bilateral PFC, PMC, and SMA recorded stronger activation in either phase or both phases of WCT compared with WMT (Fig 4C). We observed the greatest differences in Hbdiff in the left PFC (Channel 1), which presented with strongest activation during WCT, but nonsignificant activation during WMT, in early and late phases (Fig 3B and 3C). Almost all channels (excluding Channels 8 and 10 in the bilateral SMA) had higher Hbdiff during the late phase of WCT compared with during WMT (Fig 4C). The differences in Hbdiff between late-phase WCT and WMT were predominantly larger than the differences in Hbdiff between early-phase WCT and WMT (Fig 4C). These results suggested stronger and more sustained brain activation, particularly in the PFC and PMC, during dual-task performances in WCT compared with WMT.

### 3.4 Brain activity correlates with gait performance during dual tasks

Table 2 shows the brain areas associated with significant correlations (*P* < .05 with FDR correction) between early-phase Hbdiff and gait data during a specific walking condition. We observed nonsignificant correlations between brain activity and gait data during NW. By contrast, in the channels in left SMA (Channel 8) and right SMA (Channels 9 and 10), we observed significant correlations between early-phase Hbdiff and gait data during WCT (Table 2). During WMT, we predominantly observed significant correlations between early-phase Hbdiff and gait data in regions of the left PMC (Channels 3, 4, 5, and 6), with one correlation in the right SMA (Channels 10) (Table 2). In addition to the channels in bilateral SMA, channels in left PMC (Channel 5) and in right PMC (Channels 11 and 12) also exhibited significant correlations between brain activity and gait data in the late phases of WCT (Table 3). During the late phase of WMT, only the brain activation of one channel in left PMC (Channel 4) correlated with the gait performance. S2 and S3 Figs show scatter plots for the significant correlations between Hbdiff and gait data in early and late phases, respectively.

**Table 2:** Correlation analysis between the cortical Hbdiff level and gait data in the early phase (from 5 to 20 s after the task onset).

Walking condition	Brain area	Gait data	Correlation coefficient	*p*-value
**WCT**	Lt. SMA (ch.8)	cadence	0.4117	0.0040
	Lt. SMA (ch.8)	stride time	-0.4095	0.0043
	Rt. SMA (ch.9)	speed	0.4701	0.0008
	Rt. SMA (ch.9)	cadence	0.4299	0.0026
	Rt. SMA (ch.9)	stride time	-0.4466	0.0017
	Rt. SMA (ch.10)	cadence	0.4579	0.0012
	Rt. SMA (ch.10)	stride time	-0.4640	0.0004
**WMT**	Lt. PMC (ch.3)	stride length	0.4374	0.0019
	Lt. PMC (ch.4)	speed	0.5215	0.0001
	Lt. PMC (ch.4)	stride length	0.6010	<0.0001
	Lt. PMC (ch.5)	speed	0.4839	0.0005
	Lt. PMC (ch.5)	stride length	0.5148	0.0002
	Lt. PMC (ch.6)	stride length	0.4681	0.0008
	Rt. SMA (ch.10)	stride length	0.4473	0.0014

**Table 3:** Correlation analysis between the cortical Hbdiff level and gait data in the late phase (from 21 to 50 s after the task onset).

Walking condition	Brain area	Gait data	Correlation coefficient	*p*-value
**WCT**	Lt. PMC (ch.5)	speed	0.4564	0.0013
	Lt. PMC (ch.5)	cadence	0.3937	0.0062
	Lt. PMC (ch.5)	stride time	-0.4552	0.0013
	Lt. SMA (ch.8)	cadence	0.3817	0.0081
	Lt. SMA (ch.8)	stride time	-0.4391	0.0020
	Rt. SMA (ch.9)	speed	0.4818	0.0006
	Rt. SMA (ch.9)	cadence	0.4128	0.0039
	Rt. SMA (ch.9)	stride time	-0.4690	0.0009
	Rt. SMA (ch.9)	stride length	0.4308	0.0025
	Rt. SMA (ch.10)	cadence	0.4168	0.0036
	Rt. SMA (ch.10)	stride time	-0.4665	0.0009
	Rt. PMC (ch.11)	stride time	-0.3903	0.0067
	Rt. PMC (ch.12)	cadence	0.4710	0.0008
	Rt. PMC (ch.12)	stride time	-0.5135	0.0002
**WMT**	Lt. PMC (ch.4)	speed	0.5377	<0.0001
	Lt. PMC (ch.4)	stride length	0.4818	0.0005

## Discussion

This study aimed to examine the associations between cortical activation and gait performance in the presence of dual-task interference when walking. We concurrently recorded brain activities and gait data to reveal differing gait properties in participants performing NW, WCT, and WMT (Table 1), and corresponding activation of the PFC, PMC, and SMA, depending on the attentional demands or motor control required for the various conditions (Figs 3 and 4). Although we observed declines in gait performance when participants performed dual tasks, significant activation of the PMC and SMA correlated with gait data, indicating a control mechanism to maintain gait performance (Tables 2 and 3). Association between cortical activation and gait behavior enables a second task to be performed while walking (multi-tasking ability). In this study, we used a prolonged task period of 60 seconds (the common task period is 20–30 s in fNIRS motor studies), which doubled the experimental time and allowed us to investigate the temporal dynamics of brain activation in early (5–20 s) and late (21–50 s) phases during task periods.

### 4.1 Analysis of brain activation by using functional near-infrared spectroscopy

Previous studies observed similar temporal changes in hemoglobin concentration in response to various types of motor stimulation by using fNIRS. This hemodynamic response is in accordance with the mechanism of neurovascular coupling, in which neuronal activation can induce an increase in HbO concentration and a reduction in HbR concentration to facilitate local oxygenation [31, 52, 62]. Thus, either an increase in HbO or a reduction in HbR concentration can be used to identify cortical activation. In previous fNIRS studies on brain activity, HbO tended to be uniform in all participants and was a more sensitive marker of task-related cortical activation than HbR [24–27, 63, 64]. Other studies suggested that the HbR signal is minimally influenced by motion-induced changes and is thus more suitable than the HbO signal for detecting cortical activation [42]. Investigators have also used an integrated measure that combines changes in HbO and HbR, referred to as the oxygenation index, to estimate tissue oxygenation and brain activity. They proposed 2 methods for determining oxygenation: (1) the difference between HbO and HbR (ie, Hbdiff = HbO—HbR) [4, 57–59]; and (2) the oxygen saturation and tissue oxygenation index, expressed as percentages of HbO relative to total hemoglobin (ie, HbO/[HbO + HbR]) [65–67]. Although the optimal hemoglobin factor for detecting cortical activation remains under debate, we considered the dynamics of HbO and HbR simultaneously and used Hbdiff to identify brain activation. A more positive HbO value and a more negative HbR value can result in a larger Hbdiff during the task period compared with the baseline; therefore, cortical activation can be identified by an elevated Hbdiff.

### 4.2 Differences in gait properties and brain activity among dual tasks

In this study, we observed differing effects of interference from a second cognitive or motor task on gait performance. WCT and WMT caused similar and significant reductions in walking speed (Table 1), which was consistent with previous observations in a young population [8, 68]. In addition to the change in speed, several other gait properties also exhibited significant declines during WCT and WMT. During WCT, our participants focused on the 7 serial subtractions, which was a more challenging task than walking, leading to substantial decline in all gait properties (ie, reduced cadence and stride length, and increased stride time) (Table 1). During WMT, the participants intentionally altered their gait by shortening stride length to balance the bottle of water on a tray, but retained cadence and stride time (Table 1). These results suggest that passive interference by a secondary cognitive task (which is more challenging than the primary task), and an active strategy for adjusting whole-body posture to achieve stable locomotion when simultaneously performing an upper-extremity balance task, induce differences in gait performance between WCT and WMT. Evidences from previous studies also suggest that different dual-tasks diversely change gait deficits for young adults. Dual-tasks interference caused by a concurrent arithmetic task during walking led to a retardation with a range from 9.25 to 9.93% on gait velocity [15, 69], and minor dual-tasks costs ranging from 0.99% to 6.01% were reported with a concurrent manual task for young adults [5, 70, 71]. Beurskens and Bock proposed that the magnitude of gait deficits was related to demands of the concurrent task [13]. Performing tasks requiring greater cognitive and executive functions on walking, such as the serial subtraction and the stroop task, can result in higher dual-tasks costs than a concurrent motor task [13, 20].

Our results on brain oxygenation indicate cortical activation in various cerebral regions during the 3 walking conditions. In the presence of a second task, 2 specific cognitive functions, executive function and divided attention (capacity of information processing to conduct multiple tasks), are crucial to gait performance [2]. Neuroimaging studies have provided evidence to show that the PFC and its related brain networks are critical in such cognitive functions [72–74]. Our study results indicate that of all evaluated brain regions, the PFC (particularly the left PFC) exhibits the strongest continuous activation, associated with increased Hbdiff, during the entire WCT period, and tends to be associated with increased Hbdiff (though nonsignificant after FDR correction for multiple comparison) in the early phase (first 5–20 s) of NW and WMT (Fig 3). Our results on strongest activation in the PFC during WCT are supported by previous findings that the associations between cognitive functions and locomotive tasks become stronger when the locomotive task is more challenging [36, 69, 75]. The participants’ responses indicated that the 7 serial subtractions involved in WCT are more challenging than the upper-extremity balance task involved in WMT (most participants completed without dropping the bottle of water). Therefore, performing WCT can induce increased reliance and sustained demand on executive functions, as well as divided attention, leading to increased activation of the PFC to effectively allocate resources among tasks. In Fig 4C, our results also showed that almost all channels, excluding the channels 8 and 10 in the posterior SMA, exhibited significant differences in late-phase activations between the WCT and WMT conditions. These results are supported by the theory that dual tasks with higher difficulty can cause larger declines in gait performance [21], and accordingly recruit more brain activations in PMC and SMA regions to monitor and maintain the gait in a certain level.

In contrast to extensive cortical activation (in all channels) during WCT, WMT-related increases in Hbdiff were predominantly located in the SMA and PMC, with stronger activation in the early phase (Fig 3). The SMA and PMC function in the planning and initiation of complex motor activities and posture control [31, 76–78]. Increased activation in these cerebral regions might reflect demand for stabilizing the trunk and proximal limbs during locomotion [79–81]. In the healthy young adults evaluated in this study, we observed stronger activation in the SMA and PMC in the early phase of WMT (Fig 3C) to initiate sequential motion actions, which alleviated during late-phase locomotion. However, walking while performing a cognitive or upper-extremity motor task creates cognitive-motor interference with gait; therefore, skills are required to monitor and adjust whole-body posture to maintain gait performance. We observed activation of the SMA and PMC during both dual-task types, although WCT induced stronger and more continuous activation in the majority of the SMA and PMC regions compared with WMT (Fig 4C). During WMT, the right SMA (Channel 10) exhibited the highest Hbdiff compared with other channels (Figs 2C and 4), suggesting that in-phase bimanual movements are critical for balancing a bottle on a tray [82–84].

### 4.3 Brain activation maintains gait performance during dual tasks

In this study, we assessed the correlations between cerebral Hbdiff and gait performances to evaluate the interactions between brain activities and locomotive behaviors during dual tasks (Tables 2 and 3). Our combined results on gait alterations (Table 1), cerebral activities (Figs 3 and 4), and correlation analyses (Tables 2 and 3) during WCT and WMT (Table 1) indicate the role of cerebral activation in maintaining gait performance when experiencing interference from a second task. As shown in Table 1, significant reductions in walking speed and cadence occurred when participants performed WCT. As shown in Tables 2 and 3, gait properties significantly and positively correlated with cerebral Hbdiff in the PMC and SMA. These phenomena suggest that increased activation of the PMC and SMA maintains walking speed and cadence at a certain level during WCT. Although stride time increased significantly during WCT (Table 1), it negatively correlated with Hbdiff in the PMC and SMA (Tables 2 and 3), suggesting that increased cerebral activation limits increments in stride time during WCT. Conversely, during WMT, regulation of gait performance predominantly relied on activation of the left PMC (Channels 3, 4, 5, and 6) (Tables 2 and 3).

Previous gait studies without dual-task assessments reported greater activation in the left PFC and SMA during high-intensity (70% capable speed) than during low-intensity (50% and 30% capable speed) walking [24, 27]. Although participants performed highest walking speeds when NW, brain activation was nonsignificant and nonsignificantly correlated with walking speed. This is because the participants’ “work load” in this study was regulated by the presence or difficulty of a second task, resulting in reduced speed of gait. Accordingly, we observed stronger associations between brain activation and gait during challenging dual-task activities than during NW. Moreover, Harada et al. reported that the SMA activation was significantly increased during the high-intensity walking (high “work load”) and correlated with the locomotor speed and cadence, indicating that the SMA activation was associated with the gait control under a high “work load” circumstance [24]. In this study, the SMA activations played a similar role in gait control that strong correlations with gait performance were mainly observed during a difficult WCT rather than during WMT (Tables 2 and 3).

Overall, our early- and late-phase correlation analysis results indicate that cortical regions involving in modifying gait performances differ between two types of dual task. During the WCT, left SMA (Channel 8) and right SMA (Channels 9 and 10) significantly correlate with gait data in the early phase, and bilateral PMC (Channels 5, 11, and 12) is associated with the gait performance in the late phase. This extensive involvement of SMA and PMC activations in the gait adjustment may reflect a high dual-task interference during the WCT. In contrast, significant correlations between brain activations and gait data were predominantly found in left PMC (Channels 3, 4, 5, and 6) in both phases of WMT, indicating the major role of the left PMC in regulation of gait during motor dual tasks.

### 4.4 Additional considerations and potential applications

In this study, we report that dual tasks are associated with reduced gait performance and increased brain activation. These responses differ in the presence of cognitive or motor interference. Several neuropsychological theories on information processing have been proposed to explain the difficulties in performing 2 tasks simultaneously. The capacity-sharing theory and bottleneck theory presume that the functional resources of the human brain are limited in capacity [18, 19]. Therefore, performing 2 tasks with similar cognitive or motor demands causes retardations in both tasks or delays in the secondary task until the primary task has been completed. In this study, we asked participants to primarily focus on 7 serial subtractions or balancing a bottle during WCT or WMT, respectively, and observed deterioration in the secondary task (ie, gait). The theory of multiple resource models suggests that if 2 tasks use differing functional resources, dual-task interference is minimal or might not occur [16, 17]. This theory supported our results that WCT, which demands high attention and executive function, interferes with gait to a greater extent than does WMT, which recruits different resources involved in upper-extremity balancing. However, we did not record the deteriorations in performance of mental calculation or upper-extremity balance during the dual tasks compared with a single task. Therefore, we were unable to determine the effects of the dual tasks on mental calculation and upper-extremity motor task abilities.

Another limitation of this study is the lack of data from the cognitive single-task and motor single-task as comparisons. However, the brain activities during the arithmetic task or during the bimanual balance task have been well reported in literatures. Several neuroimaging studies reported that a reproducible set of prefrontal, parietal, and cingulate areas was activated during a calculation task [85–88]. Although all channels in PFC, SMA, and PMC exhibited significant brain activations (Fig 4A), the Hbdiff in PMC and SMA regions showed significant correlations with gait performance during the WCT (Tables 2 and 3), suggesting PMC and SMA were associated with the gait adjustment rather than the cognitive task itself. Several studies reported that the bimanual in-phase movements involved in our balance tasks were highly correlated to the activation in the SMA [82–84]. Our results also confirmed strong activations in SMA channels during the WMT (Fig 4B). However, the significant correlations between brain activations and gait data were predominantly found in left PMC rather than SMA regions suggesting that left PMC is critical in the gait control under the motor dual-task interference.

Several factors, including aging and diseases of the central nervous system, can affect gait performance during attention- or motor-demanding locomotive tasks. Previous studies assessed healthy elderly people and young adults performing dual tasks and observed greater reduction in gait speed and inferior performance in the second task in the healthy elderly people compared with in the young adults [1, 5–8]. Studies have also observed that gait disturbance during dual tasks increases in patients with neurodegenerative disorders, stroke, and brain injuries. Patients with Parkinson’s or Alzheimer’s disease exhibit slower walking speeds, increased gait variability, and asymmetry compared with healthy elderly people during dual tasks [89–94]. However, dual-task interference in elderly people can be alleviated by providing appropriate training or medication for ≥3 months [95–97]. Our results indicate that simultaneous measures of cortical activation and gait performance could be helpful for determining the effects of aging or neuronal diseases on gait control. The effects of rehabilitation and training could also potentially be evaluated using dual-task assessments in future studies.

### 4.5 Conclusion

In conclusion, this study collected brain activity and gait data to elucidate the changes in cortical activation and gait performance when healthy young adults perform dual tasks. The fNIRS technique overcame the limitations of conventional neuroimaging tools for monitoring brain activity during locomotive tasks. Our study results indicate that the activities of the PFC, PMC, and SMA alter according to the demands of individual tasks, and that activation of these brain regions maintains gait performance at a certain level when performing a second task while walking. In future studies, we aim to further examine the relationships between cortical regulation and locomotive behaviors in various populations to evaluate the effects of aging and neurological diseases on dual tasks.

## Supporting Information

S1 Fig A diagram of coregistration procedure for unifying the coordinate system of fNIRS optodes and brain anatomy. (a) The anatomical landmarks, including nasion and bilateral pre-auricles, and surface locations of fNIRS optodes were recorded using a 3D digitizer with ultrasound transmitters and sensors. (b) The three anatomical landmarks were also defined manually by a radiological technician (CF Lu) on the participant’s surface of MR structural images. (c) The cerebral cortex image was further extracted from T1-weighted images using the segmentation algorithm of SPM8 software (http://www.fil.ion.ucl.ac.uk/spm/). (d) We unified the 3D coordinate system by coregistrating two sets of landmarks defined on separately on real surface in (a) and MR images in (b) using the iterative closest point algorithm. The coordinates of the fNIRS optodes were accordingly transferred into the space of MR images. Finally, the integrated visualization of head surface, cortical surface and locations of fNIRS optodes can be used to check if the fNIRS channels were located in the target regions, namely, PFC, PMC, and SMA for each participant.
(DOC)

S2 Fig The scatter plots for the significant correlations (*p*<0.05 with FDR correction) between the gait data and the cortical Hbdiff level in the early phases of (a) WCT and (b) WMT. Each circle represents the measurement of a task block.
(DOC)

S3 Fig The scatter plots for the significant correlations (*p*<0.05 with FDR correction) between the gait data and the cortical Hbdiff level in the late phases of (a) WCT and (b) WMT. Each circle represents the measurement of a task block.
(DOC)
