# Supplementary material for: Maintaining Gait Performance by Cortical Activation during Dual-Task Interference: A Functional Near-Infrared Spectroscopy Study
Source: PLoS One. 2015 Jun 16;10(6):e0129390. doi: 10.1371/journal.pone.0129390 (PMC4469417; doi:10.1371/journal.pone.0129390)
Supplement: S1 Fig — (a) The anatomical landmarks, including nasion and bilateral pre-auricles, and surface locations of fNIRS optodes were recorded using a 3D digitizer with ultrasound transmitters and sensors. (b) The three anatomical landmarks were also defined manually by a radiological technician (CF Lu) on the participant’s surface of MR structural images. (c) The cerebral cortex image was further extracted from T1-weighted images using the segmentation algorithm of SPM8 software (http://www.fil.ion.ucl.ac.uk/spm/). (d) We unified the 3D coordinate system by coregistrating two sets of landmarks defined on separately on real surface in (a) and MR images in (b) using the iterative closest point algorithm. The coordinates of the fNIRS optodes were accordingly transferred into the space of MR images. Finally, the integrated visualization of head surface, cortical surface and locations of fNIRS optodes can be used to check if the fNIRS channels were located in the target regions, namely, PFC, PMC, and SMA for each participant. (DOC) [file pone.0129390.s001.doc]

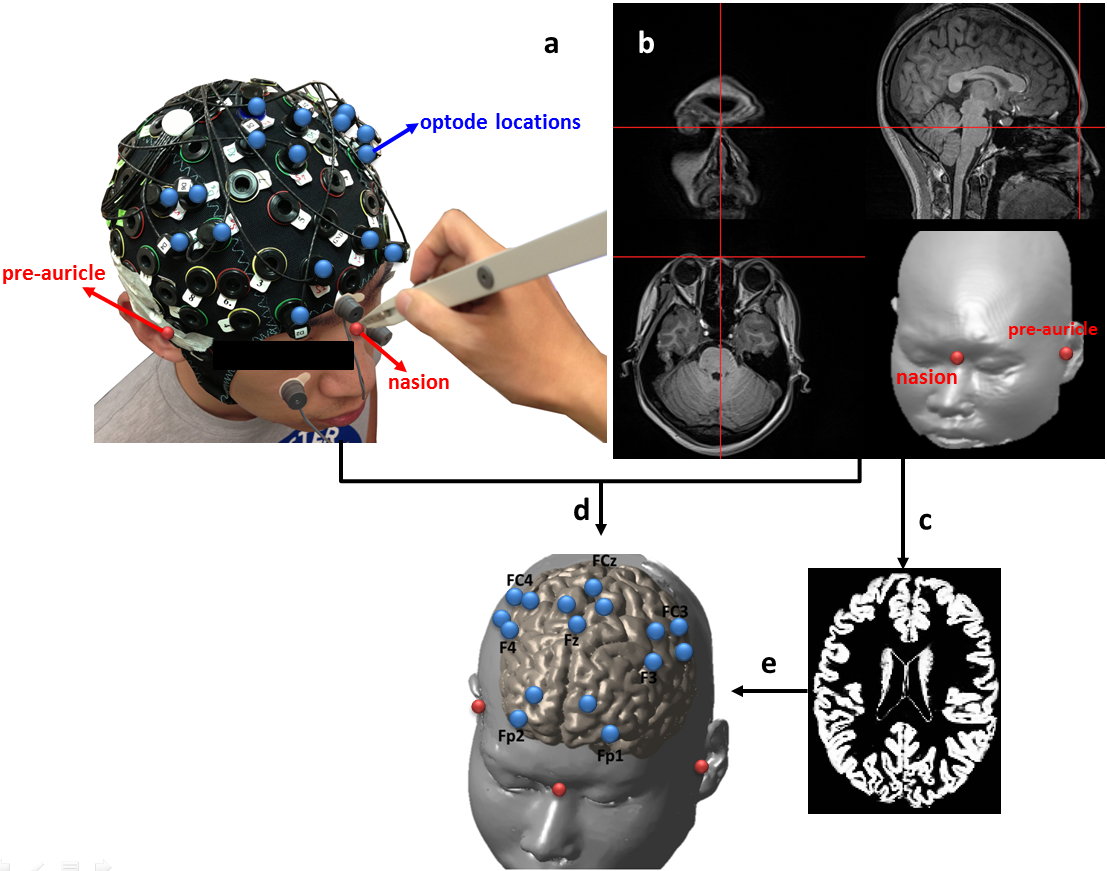


**Figure S1. A diagram of coregistration procedure for unifying the coordinate system of fNIRS optodes and brain anatomy.** (a) The anatomical landmarks, including nasion and bilateral pre-auricles, and surface locations of fNIRS optodes were recorded using a 3D digitizer with ultrasound transmitters and sensors. (b) The three anatomical landmarks were also defined manually by a radiological technician (CF Lu) on the participant’s surface of MR structural images. (c) The cerebral cortex image was further extracted from T1-weighted images using the segmentation algorithm of SPM8 software (<http://www.fil.ion.ucl.ac.uk/spm/>). (d) We unified the 3D coordinate system by coregistrating two sets of landmarks defined on separately on real surface in (a) and MR images in (b) using the iterative closest point algorithm. The coordinates of the fNIRS optodes were accordingly transferred into the space of MR images. Finally, the integrated visualization of head surface, cortical surface and locations of fNIRS optodes can be used to check if the fNIRS channels were located in the target regions, namely, PFC, PMC, and SMA for each participant.
