# Supplementary material for: Maintaining Gait Performance by Cortical Activation during Dual-Task Interference: A Functional Near-Infrared Spectroscopy Study
Source: PLoS One. 2015 Jun 16;10(6):e0129390. doi: 10.1371/journal.pone.0129390 (PMC4469417; doi:10.1371/journal.pone.0129390)
Supplement: S3 Fig — Each circle represents the measurement of a task block. (DOC) [file pone.0129390.s003.doc]

**
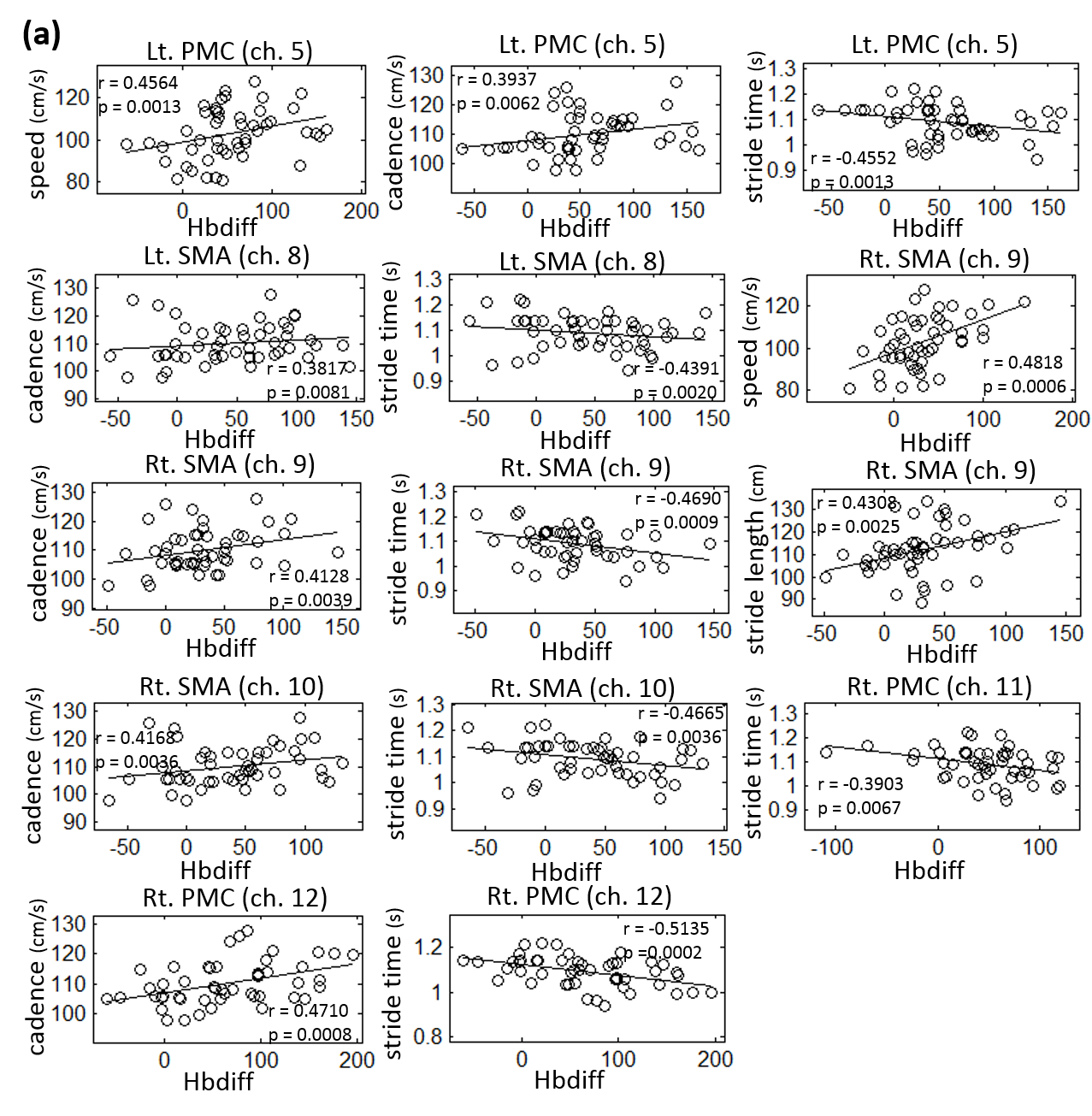
**

**
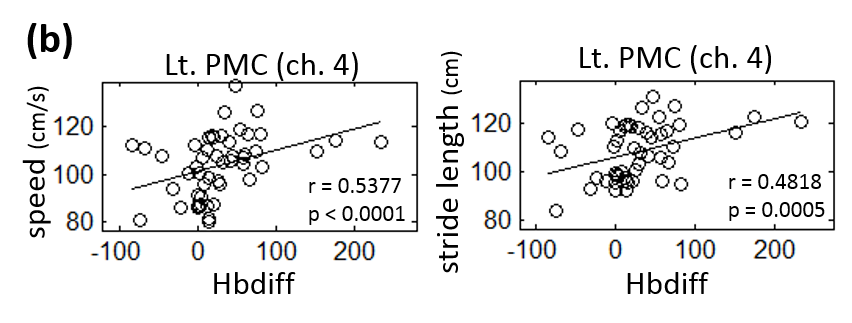
**

**Figure S3 The scatter plots for the significant correlations (*p*<0.05 with FDR correction) between the gait data and the cortical Hbdiff level in the late phases of (a) WCT and (b) WMT.** Each circle represents the measurement of a task block.
